# 95923 TBI-on-a-chip: Linking physical impact to neurodegeneration by decrypting primary and secondary injury mechanisms

**DOI:** 10.1017/cts.2021.636

**Published:** 2021-03-31

**Authors:** Edmond Rogers, Timothy Beauclair, Guenter Gross, Riyi Shi

**Affiliations:** 1 Purdue University; 2 University of North Texas

## Abstract

ABSTRACT IMPACT: This unique approach has the capability to elucidate the pathological mechanisms underlying traumatic brain injuries and neurodegeneration, both separately and in concert, while simultaneously providing a semi-high throughput model for investigating potential pharmaceutical interventions: discoveries that would have major translational implications and a significant impact worldwide. OBJECTIVES/GOALS: We aim to improve our understanding of the mechanisms behind the development of neurodegenerative diseases by utilizing the link between traumatic brain injuries and demonstrated biomarkers with our innovative TBI on a chip model. With this tool, we hope to provide new pathological insights and explore potential pharmaceutical interventions. METHODS/STUDY POPULATION: E16 murine cortical networks were cultured onto reusable, optically transparent MEAs (fabricated in-house), and subjected to a clinically-relevant range (30-300g) of impact g-forces, utilizing our exciting new in vitro model of trauma (TBI on a chip) with real-time electrophysiological and morphological access. Impacts were systematically applied at varying intensities, repetitions, and time points, and fixed 24-hours post. Basic immunocytochemical techniques were used to investigate post-impact levels of acrolein, an established biomarker of both post-TBI oxidative stress and neurodegeneration (ND), and compared to procedurally and age-matched non-impact control networks. In addition, several other TBI/ND biomarker investigations are in progress (βA42, α-synuclein, and phosphorylated tau). RESULTS/ANTICIPATED RESULTS: Impact experiments revealed significant, force-dependent increases of acrolein (acrolein-lysine adducts) at 24hrs post impact, indicative of impact-linked neuronal degeneration. These changes were amplified by the following manipulations: increasing g-force exposure (30-250 g); the rapid (4-6 sec interval) application of multiple impacts (1, 3, 5 and 10x); and exposure to 40 mM EtOH (10 min duration immediately following impact). Further, we demonstrate the enhancement of injury recovery as a function of: increasing time intervals between repeated hits; Hydralazine exposure. In addition, conditioned media from maximally-impacted cultures can cause acrolein elevation when introduced to non-impact, control networks, indicating acrolein’s role as a diffusive-factor in post-TBI secondary injuries. DISCUSSION/SIGNIFICANCE OF FINDINGS: This novel approach provides unprecedented resolution, and is improving our understanding of the pathological mechanisms underlying both TBI and ND. Combined with our established in vivo models of trauma and computer modeling, we hope to better guide our translational laboratory endeavors and help improve clinical diagnoses and treatments.

